# Dr. M. Marotte on Emetics in Confirmed Croup

**Published:** 1842-07-23

**Authors:** 


*Emetics in Confirmed Croup.*—M. Marotte, in the "Gazette Médicale," enforces the necessity of repeated and large doses of emetics, aided by local depletion, mild purgatives, and blisters, in decided cases of croup. Dr. Marotte agrees with Dr. Delaroque on the necessity of acting vigorously within the first two hours from the accession of the disease: thus, he commences by applying leeches, and whilst the patient is in a state bordering on syncope, he gives large doses of emetics, being indifferent as to the particular emetic administered, and follows them up with a blister, all within an hour or an hour and a half. If the first administration of emetics only produce temporary relief, they are to be repeated every three, four, or five hours, until a decided benefit is obtained.—*Ibid.*

